# Complete genome of *Flavobacterium pectinovorum* str. ZE23VCel01 obtained through Nanopore Q20+ chemistry

**DOI:** 10.1128/mra.00715-23

**Published:** 2023-12-06

**Authors:** Lucas Serra Moncadas, Vanessa Schnellmann, Cyrill Hofer, Angel Rain-Franco, Adrian-Stefan Andrei

**Affiliations:** 1 Microbial Evogenomics Lab, Limnological Station, Department of Plant and Microbial Biology, University of Zurich, Kilchberg, Switzerland; 2 Faculty of Sciences, University of Zurich, Zurich, Switzerland; 3 Limnological Station, Department of Plant and Microbial Biology, University of Zurich, Kilchberg, Switzerland; Queens College, Queens, New York, USA

**Keywords:** genomics, DNA sequencing, Nanopore, *Flavobacterium*

## Abstract

This study reports the complete genome of *Flavobacterium pectinovorum* str. ZE23VCel01 isolated from a freshwater environment. By means of Nanopore Q20+ chemistry, the chromosome was assembled as a circular element with a length of 5.9 Mbp, a GC content of 33.58%, and a coverage of 122×.

## ANNOUNCEMENT

Members within the *Flavobacterium* genus primarily occupy freshwater environments and hold significant ecological relevance, as they are accountable for serious diseases in freshwater fishes. By means of Nanopore Q20+ technology, it is possible to gain a novel perspective on their biological features and ecological impact on aquatic environments (1).

The isolated strain from the surface waters of Lake Zurich (47°18′N, 8°34′E, Switzerland) was cultivated in agar plates (15 g/L) supplemented with artificial lake water (2) using cellobiose as a carbon source (100 µM final concentration). After 21 days of cultivation, colonies were purified through sequential streaking cycles and cryopreserved as described elsewhere (2). The isolated strain was reactivated in artificial lake water supplemented with glucose (100 µM) for 1 week and grown in commercial LB medium (Difco, 240210) for 2 days. From the resuscitated culture (3), DNA extraction was performed using the Quick-DNA HMW MagBead kit (Zymo Research). The obtained DNA was purified with Beckman Coulter AMPure XP magnetic beads and subsequently loaded on a FLO-MIN114 (R10.4.1) new chemistry flow cell and used for sequencing on a Nanopore minION Mk1B. The sequencing 1D library was constructed with the SQK-NBD114.24 Native Barcoding Kit 24 V14 (ONT, Oxford, UK) in conformity with the manufacturer’s instructions and long DNA fragment selection (without any prior DNA fragmentation).

Sequencing resulted in 1,108,124 raw reads (mean read length 3.9 kbp; median read quality 14.3; N50 5.4 kbp) that were basecalled with Guppy v.6.4.6 (superaccurate basecalling, 400 bp) before quality filtration with chopper v.0.2.0 (4). A minimum threshold of Q17 was used, followed by the removal of the first 10 bases and reads < 1,000 bp (164,407 reads; mean read length 4.5 kbp; median read quality 17.9; N50 5.5 kbp). Assembly was conducted with Flye 2.9.2-b1786 (5) (-nano—corr option), leading to a circular chromosome (5,936,092 bp). The obtained circular genome was subsequently classified using GTDB-Tk v2.2.6 (6) combined with a comparison of its 16S rRNA gene (predicted with barrnap 0.9) against the SILVA database (v138.1) (7). Genome completeness (99.22%) and contamination (1.88%) were assessed with CheckM v1.1.3 (8). Prokka v1.12 (9) and NCBI’s PGAP pipeline (10) were used to predict coding DNA sequences and tRNAs (4,970 genes; 30 pseudo-genes). BlastKOALA (11) was used to assign KO identifiers to orthologous genes. Metabolic reconstruction and general biological functions were conducted with the online KEGG mapping tools using the previously obtained KO numbers. PFAM domains were identified in the proteome using the script pfam_scan.pl with the PFAM database release 32 (12). Default parameters were used except where otherwise specified.

Taxonomical classification suggests that the genome belongs to a new *Flavobacterium pectinovorum* strain by GTDB v214.1 (95.17% similarity to GCA_900142715, 98.13% coverage) and SILVA v138.1 (98.87% similarity) databases. The genome-inferred metabolic reconstructions pictured a microorganism with the metabolic capability to synthesize most of the proteinogenic amino acids apart from arginine.

## Data Availability

Sequence data are available through the National Center for Biotechnology Information (NCBI) via the BioProject PRJNA991734 (Biosample SAMN36315996, Accession GCA_030505355.1).
